# In sight on olive oil maceration and supercritical CO_2_ in extracting rosemary essential oil

**DOI:** 10.1038/s41598-024-79832-y

**Published:** 2024-11-29

**Authors:** Maha Mohamed Soltan, Sabry Mahfouz, Fatma H. Motawe, Eman A. Karam, Ali El-Hagrassi

**Affiliations:** 1 Biology Unit, Central Laboratory for Pharmaceutical and Drug Industries Research Institute, Chemistry of Medicinal Plants Department, National Research Centre, El-Buhouth St. 33, Dokki, Cairo, 12622 Egypt; 2 Medicinal and Aromatic Plant Research Department, National Research Centre, El-Buhouth St. 33, Dokki, Cairo, 12622 Egypt; 3 Microbial Chemistry Department, National Research Centre, El-Buhouth St. 33, Dokki, Cairo, 12622 Egypt; 4 Phytochemistry and Plant Systematics Department, National Research Centre, El-Buhouth St. 33, Dokki, Cairo, 12622 Egypt

**Keywords:** Hydrodistillation, Maceration, Supercritical CO_2_, Green chemistry, Antimicrobial, Chromatography, Drug discovery, Microbiology, Plant sciences

## Abstract

The flavor, nutritional, and medicinal value of rosemary are well known. Hydrodistilled (HD) essential oil was prepared in the present study as a standard. Olive oil maceration (OM) and supercritical carbon dioxide (SC-CO_2_) technology were separately applied to extract the essential oil of rosemary. The three obtained products, HD, OM, and SC-CO_2_, were compared concerning their main constituents using GC‒MS. Their antimicrobial properties were evaluated against eight microbes by the disc diffusion assay. Interestingly, both 1,8-cineol and camphor were the major compounds in the three oils. α-Pinene was also detected in large amounts in both HD and OM. Additionally, borneol was the third major component in SC-CO_2_. The antimicrobial results revealed differential effects against six microbes. However, HD oil also exhibited antifungal activity. Maceration is a green extraction procedure that is easy to perform in households, and attention must be paid to olive oil maceration as a complementary medicine that originated in pharaohs.

## Introduction

When food substances are accessible in their active forms, they protect our bodies from disease attacks, in addition to their nutritional value. *Rosmarinus officinalis* (rosemary) is popular in Egyptian herbal shops (Attars) as dry herb or its essential oil form. It is also available fresh in vegetarian supermarkets. The plant is a medicinal herb belonging to the Lamiaceae family. It is categorized as one of the spices with the highest antioxidant activity^1^. Its antimicrobial properties have also been reported^2^. Both herbs and fresh plants are widely used as preservatives for flavoring foods and for treating various illnesses^3,4^. It is commonly used in industry for aromatizing olive oil^5^. In the last decade, ultrasonic extraction of rosemary has been further investigated. However, to the best of our knowledge, no scientific reports have shown the impact of traditional maceration on the bioactivity of chemical constituents. Maceration, the process of releasing plant essences into a fixed oil, originated in ancient Egypt^6^. In the present study, the dried herb was subjected to hydrodistillation (HD) or olive oil maceration (OM) using a household-friendly procedure without the need for laboratory tools such as vortexing or ultrasonication. Supercritical carbon dioxide (SC-CO_2_) technology was also applied. The products from the three procedures were compared concerning their main contents of essential oil and weather impacting their antimicrobial activity. The antimicrobial potential of natural extracts and oils is an alternative to antibiotics, particularly when they are edible and have a prolonged guarantee in traditional medicine. The massive use of antibiotics promotes the resistance of pathogenic bacteria, resulting in increased doses of antibiotics^7^. Scientific communities need more investigations to fill the gap between bioactive profiles and dosing. Thus, in the present study, the antimicrobial efficacy of the tested rosemary oils was investigated against eight microbes. To the best of our knowledge, this comparison has not been addressed before. In addition, the conditions of SC-CO_2_ and maceration with olive oil have not been sufficiently investigated.

## Materials and methods

### Plant materials

Rosemary dry leaves are commercially available in local herbal markets (Attar) and are provided and regularly hydrodistilled through the unit of pressing and extraction of natural oils, National Research Centre, Egypt.

### Oil extraction methods

Hydrodistillation, maceration in cold press olive oil, and SC-CO_2_ were used to extract the essential oils of interest.

### Hydrodistillation

Hydrodistillation was the selected technique for preparing the essential oil of rosemary to obtain the highest oil yield^8^. This process involves soaking plant material in a container filled with water and heat. The distillation container is made of glass and has a condensing apparatus attached to a receiving flask. The steam and oil vapor produced as a result of the heating source are condensed. Finally, the product was collected by a Clevenger-type apparatus.

### Maceration

Interestingly, maceration of olive oil was practiced by ancient Egyptians. It was prescribed for the famous medical Ebers’ Papyrus^9^. Maceration was performed in the present study by soaking 75 g of powdered rosemary in a glass container containing cold pressed olive oil. Actually, 300 ml of the olive oil was enough to steep the 75 g rosemary powdered herb while slowly adding a certain portion of the oil up to obvious penetration then after adding another portion up to 300 ml.

The container was kept dry in the dark for 24 days at room temperature (27–31 °C). The ambient temperatures were recorded while the mixture was gently mixed well every two days and kept again, in dark. At the indicated time, the contents were poured into clean dry cotton gauze, filtered, and collected in a brown dry glass container, which was then kept in a refrigerator prior to chemical and biological evaluation. The residue from the herbs in gauze was separated and washed with another 200 ml of olive oil for half an hour, and the process of removing most of the oil was repeated twice by pressing the gauze to wash out the contacted portions of macerated olive oil still on the surface of rosemary residues. The residue was taken and subjected to hydrodistillation to determine the unextracted essential oil portion after maceration (unmacerated quantity). For biological evaluation, the oil was filtered with Whatman No. 3 (10004150).

### ***SC-CO***_***2***_*** extraction***

The process of using CO_2_ in the food industry began in the 1970s. As a result, the importance of, *e.g.,* caffeine separation and refining of other plant components has been highlighted. It has been increasingly used because it is a green solution, particularly for the extraction of natural compounds^10^. SC-CO_2_ extraction was performed using a laboratory-scale unit at the National Research Centre (Spe-ed TM SFE-2/2, Applied separations, Built in conjunction with the USDA1- USA). The extraction vessel was loaded with approximately 77 g of the coarsely powdered rosemary aerial parts. The static technique at 40 °C and 100 bar pressure was first performed, followed by dynamic extraction by elevating the pressure to 100, 200 and 300 bar. The time was 2 h for each interval pressure at a CO_2_ mass flow rate of 1.0 g/min^11,12^.

### Chemical analysis

#### Gas chromatography–mass spectrometry (GC–MS) analysis

This technique was used to analyze the essential oils. The GC‒MS system (Agilent Technologies) was equipped with a gas chromatograph (7890B) and mass spectrometer detector (5977A) at the Central Laboratories Network, National Research Centre, Cairo, Egypt. The samples were diluted with hexane (1:19, v/v). The GC was equipped with an HP-5MS column (30 m × 0.25 mm internal diameter and 0.25 μM film thickness). Analyses were carried out using helium as the carrier gas at a flow rate of 1.0 ml/min at a split ratio of 1:30, an injection volume of 1 µl and the following temperature program: 40 °C for 1 min, increasing at 4 °C/min to 150 °C and held for 6 min, and increasing at 4 °C/min to 210 °C and held for 1 min. The injector and detector were held at 280 °C and 220 °C, respectively. Mass spectra were obtained by electron ionization (EI) at 70 eV using a spectral range of m/z 50–550 and a solvent delay of 3 min. Identification of different constituents was determined by comparing the spectrum fragmentation pattern with those stored in Wiley and NIST Mass Spectral Library data.

#### Gas chromatography (GC)

It was used to determine the fatty acid composition of the fixed oils. The GC model 7890B from Agilent Technologies was equipped with a flame ionization detector at the Central Laboratories Network, National Research Centre, Cairo, Egypt. Separation was achieved using a DB-Wax column (60 m × 0.25 mm internal diameter and 0.25 μm film thickness). Analyses were carried out using helium as the carrier gas at a flow rate of 2.1 ml/min in split-less mode, with an injection volume of 1 µl and the following temperature program: 50 °C for 1 min; increase at 25 °C/min to 175 °C; increase at 4 °C/min to 235 °C; and hold for 20 min. The injector and detector were held at 260 °C and 280 °C, respectively.

### Antimicrobial investigations

#### Microorganisms

All the extracted oils were screened against 8 microorganisms, namely, two gram-positive bacteria (*Staphylococcus aureus* ATCC 25923 *and Bacillus subtilis* 6633), two gram-negative bacteria (*Escherichia coli* ATCC 8739 and *Salmonella typhimurium* ATCC 14028), two yeast-like fungi (*Candida albicans* ATCC 10231 *and Candida tropicalis* ATCC 750), two fungi (*Aspergillus niger* EM77 (KF774181)), *and Macrophomina phaseolina* NRRL A62743.

#### Antimicrobial assay

The antimicrobial properties of the tested oils were determined by the agar diffusion technique^13^**.** In brief, sterile nutrient that supports the growth of various types of bacteria^14^ and Czapek’s dox agar media; contained sodium nitrate as the sole source of nitrogen in cultivating fungi^15^ were freshly prepared**.** An inoculum of 1 × 10^5^ CFU/mL from each microbe was separately, adjusted while serially diluting each microbe cell suspension. 50 μl of each inoculum was edited to the corresponding prepared medium and poured into 10 cm diameter Petri dishes. Preliminary experiments were performed to determine the optimum concentration applied to the discs. This process resulted in a total volume of 10 μl (2 μl of EO mixed in 8 μl of olive oil). Ten microliters of each sample were placed on a filter paper disc (0.5 cm diameter). After depositing all the prepared discs onto the surface of the inoculated agar plates and maintaining the plates at a low temperature of 4 °C for two hours prior to incubation, diffusion favored microbial growth, as indicated by the diameter of the inhibition zone in mm. Amoxicillin trihydrate (10 µg/ml), Epico (amoxicillin) was the standard antibacterial agent, while clotrimazole (1 mg/ml) and ADCO (Candistan) were used during the antifungal assays. The plates were incubated 24 h. at 35 °C for bacteria. In the other hand, plates of yeasts and fungi were incubated 24 and 72 h., respectively at 30 °C. The previous conditions were previously reported by Soltan et., al. (2020)^16^.

## Results and discussion

### Preparation of rosemary essential oil

Hydrodistillation (HD) is a traditional standard technique for extracting essential oils^17^. In the present study, it was used as a standard to evaluate both olive oil maceration (OM) and supercritical carbon dioxide (SC-CO_2_) techniques for extracting rosemary bioactive ingredients. An antimicrobial disc diffusion assay was used to investigate the impact of differences in chemical constituents on the susceptibility of the selected microbes as well as their growth inhibition.

Regarding the maceration technique, cold-pressed virgin olive oil (total acidity of 0.5%; peroxide value of 13.5 meq/kg) was used for extracting rosemary oil constituents due to its great benefits in both pharmaceuticals and nutraceuticals^18^. The unmacerated rosemary EO quantity was recorded 0.42 ml/100 g herb. By omitting this residue volume (0.42 ml/100 g herb) from the standard HD volume (1.4 ml/100 g herb), we obtained the real macerated volume. Table 1 shows that the yield of rosemary OM was 0.98 ml/100 g herb, representing 70% of that obtained by HD (1.4 ml/100 g herb). Notably, trials involving obtaining the volatile constituents of rosemary were performed without stirring the mixture. As a result, we obtained only 15% of the expected EO relative to HD. For SC-CO_2_, 1.3% of the extract relative to the applied dry weight of rosemary was collected. The extract was paste-like and insoluble in methanol but soluble in chloroform.

**Table 1:** Yield of the extracted essential oils of rosemary.

Technique	Yield /100 g herb	% Relative to HD
HD	1.4 ml	100%
OM	0.98 ml	70%
SC-CO_2_	1.3 g	ND

HD, hydrodistillation; OM, olive oil maceration; SC-CO_2_, supercritical carbon dioxide; ND, not determined due to the yield was a paste-like extract and not liquified oil; %, percentage.

### Chemical analysis

#### Fatty acid composition of olive oil before and after rosemary maceration

**Fig. 1:**
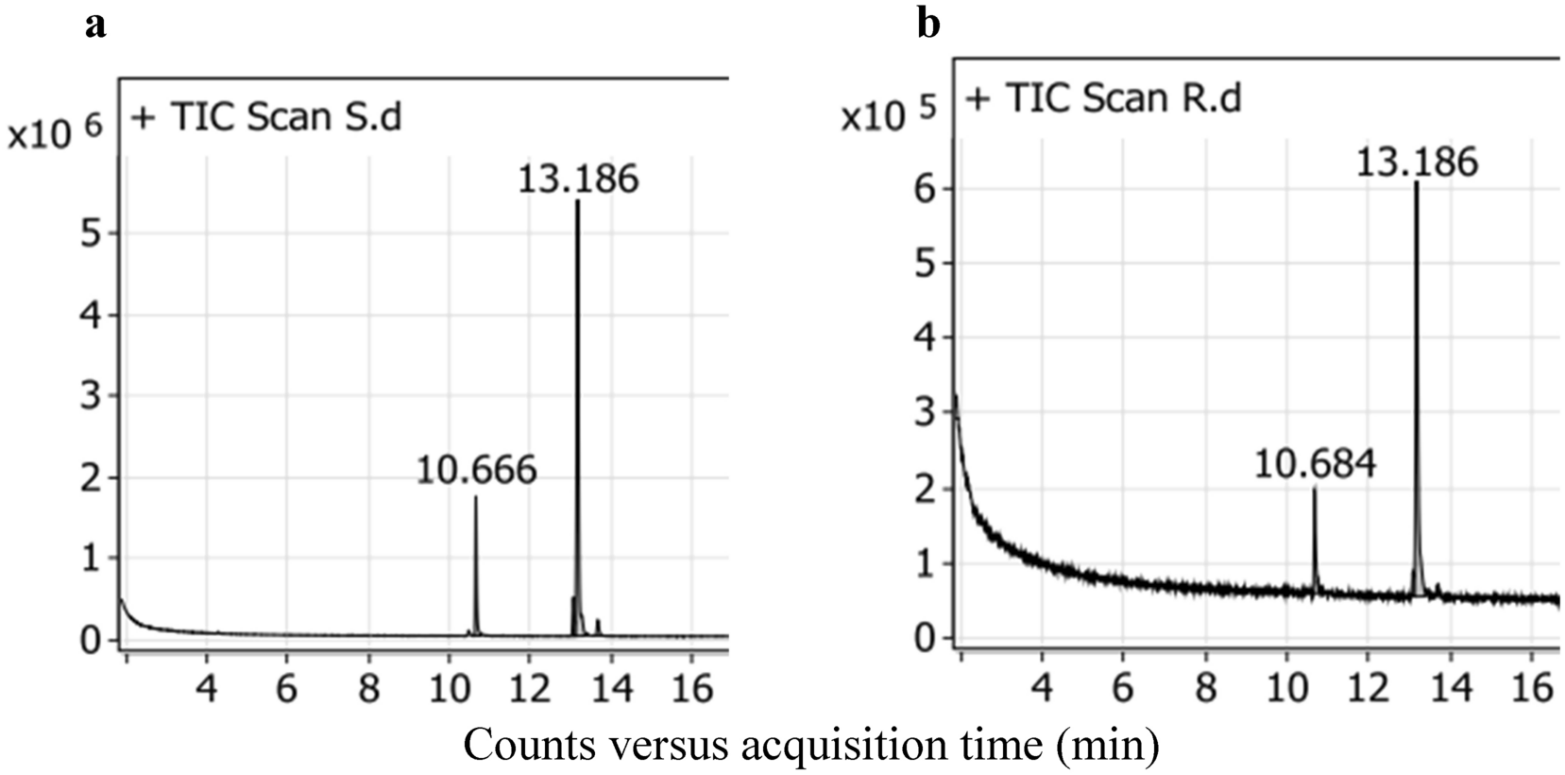
Main fatty acids of olive oils. Olive oil macerated with rosemary oil (**a**) and pure olive oil (**b**).

Extra virgin olive oil is enriched with a beneficial fatty acid profile^19^. The pure olive oil was subjected to gas chromatography before and after rosemary maceration. The main fatty acid composition is illustrated in Table 2 and Fig. 1. The chemical profile of the olive oil showed approximately the same percentage of saturated fatty acids (SFA) before and after maceration. However, a slight increase was recorded after maceration because the rosemary itself contains stearic and palmitic fatty acids^20^. On the other hand, the monounsaturated fatty acid (MUFA) content was normal and was 79.52% and 75.85% in pure olive oil and macerated oil, respectively. In the present study, the content of linoleic acid, a polyunsaturated fatty acid (PUFA), in OM was twice that in pure olive oil**.** Our results are explained by the findings of Elbanna et al. (2018)^20^. Their cold-pressed rosemary oil has been reported to contain linoleic (41.7%) and oleic (41.2%) acids as the major fatty acids. Generally, oleic and linoleic acids are highly important fatty acids that are valued in oils for both edible and nutraceutical purposes^21^.

**Table 2:** Main fatty acid composition of olive oil used for maceration.

Fatty acids	Rosemary/Olive (OM)		Olive	
	Rt (min)	Area (%)	Rt (min)	Area (%)
Palmitic acid (C16:0)	10.666	15.46	10.684	15.34
Linoleic acid (C18:2)	13.077	5.65	13.101	2.65
Oleic acid (C18:1)	13.186	75.85	13.186	79.52
Stearic acid (C18:0)	13.681	3.04	13.699	2.49
Ʃ SFA		18.50		17.83
Ʃ USFA		81.50		82.17

OM, olive oil maceration; Rt, retention time; min, minute; %, percentage; SFA, saturated fatty acid; USFA, unsaturated fatty acid.

### GC-M analysis of the three prepared rosemary oils

**Fig. 2:**
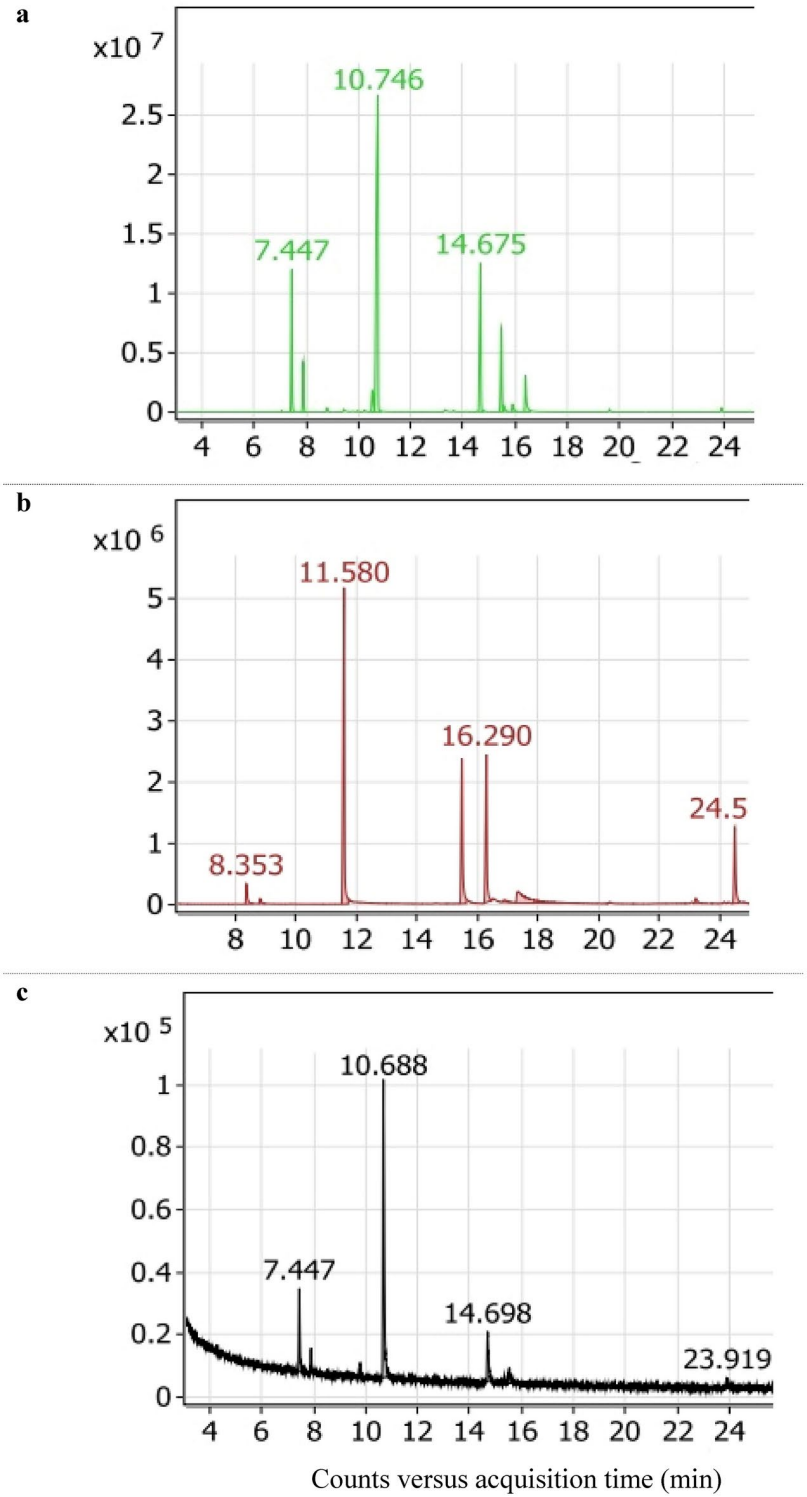
Essential oil composition of rosemary essential oils extracted by hydrodistillation (**a**), SC-CO_2_ (**b**) or olive oil maceration (**c**).

The analyzed volatile contents of the three prepared products (HD, SC-CO_2_, and OM) measured by GC‒MS are displayed in Table 3, while their chromatograms are given in Fig. 2. From a chemical point of view, rosemary essential oil is classified into three main chemotypes: cineoliferum (a high percentage of 1,8-cineole (Eucalyptol), camphoriferum (camphor is more than 20%), and verbenoniferum (verbenone is more than 15%)^22^. In the present study, the monoterpene 1,8-cineol was the most abundant of the three analyzed rosemary volatile contents. It means that the investigated rosemary was cineoliferum chemotype.

**Table 3:** Chemical composition of rosemary essential oil extracted by hydrodistillation, supercritical CO_2_ and olive oil maceration.

Compound	Hydro distillation		Supercritical CO_2_		Maceration	
	Rt (min)	Area (%)	Rt (min)	Area (%)	Rt (min)	Area (%)
α-Pinene	7.447	11.38	8.353	2.68	7.447	14.44
Camphene	7.884	4.16	8.811	0.75	7.896	4.14
*p*-Cymene	10.531	2.69				
1,8-cineole	10.746	50.60	11.58	37.54	10.688	62.67
Camphor	14.675	16.55	15.483	18.51	14.698	12.81
Borneol	15.473	8.52	16.290	18.79	15.526	3.75
6-Terpineol	15.561	0.67				
4-Terpineol	15.905	1.05				
α-Terpinol	16.394	4.38	17.32	10.77	15.99	0.50
Copaene			23.219	1.03		
Caryophyllene			24.512	9.94	23.92	1.70

Rt, retention time; min, minute; %, percentage; –, not detected. Measurements were performed twice, but α-terpinol was detected only once during the maceration procedure.

In the present results, we found that two compounds were principles in the three essential oils: 1,8-cineole (50.60%, 37.54%, and 62.67% in the HD, SC-CO_2_, and OM volatile contents, respectively), and camphor (16.55%, 18.51%, and 12.81% in the HD, SC-CO_2_, and OM volatile contents, respectively). Further two compounds were represented in considerable amounts; α-pinene (11.38%, 14.44% in the HD and OM volatile contents, respectively) and borneol (8.52%, 18.79% in the HD, and SC-CO_2_ volatile contents, respectively_)_. Among the three analyzed rosemary products, SC-CO_2_ contained the highest amount of borneol (18.79%). In contrast, OM had the least amount (3.75%) of the later monoterpene. In addition to higher amounts of α-terpinol (10.77%) and caryophyllene (10.77%) were determined in the SC-CO_2_ oil rather than HD and OM oils. The low-molecular-weight molecules were detected as minor constituents in SC-CO_2_ oil. This finding was in agreement with a previously published report^23^. Notably, three monoterpenes, p-cymene**,** 4-terpineol**,** and 6-terpineol, were detected only in the HD oil.

Interestingly and like the HD, the OM oil had approximately, the same proportions of α-pinene and camphene (0.23 and 0.07, respectively) relative to 1,8-cineole (Table 4). On the other hand, the proportions of camphor relative to 1,8-cineol were 0.33 in HD and 0.20 in OM. Furthermore, camphor and borneol relative to the major molecule; 1,8-cineole were equally represented by 0.5 while α-terpinol was 0.29 in the SC-CO_2_ oil (Table 4).

**Table 4:** Main components and their proportions to 1,8-cineole of rosemary essential oils extracted by hydrodistillation, supercritical CO_2_ and olive oil maceration.

Compound	Hydro distillation	Supercritical CO_2_	Maceration
α-Pinene	0.22	0.07	0.23
Camphene	0.08	0.02	0.07
1,8-cineole	1.00	1.00	1.00
Camphor	0.33	0.49	0.20
Borneol	0.17	0.50	0.06
α-Terpinol	0.09	0.29	0.03
Caryophyllene	–	0.26	0.03

### Antimicrobial evaluation of the extracted essential oils

Essential oils are quickly evaporated and are better mixed with a fixed oil as a carrier. Olive oil is a good carrier; however, it is also known for its antimicrobial properties^24^. We performed a titration to select a suitable volume of olive oil as a carrier. Titration resulted in 8 µl/disc of olive oil, which was the maximum volume that did not show an antimicrobial effect against the selected panel of microbes. The maximum volume to saturate the discs (0.5 cm diameter) prior to being incubated with the microbes was 10 µl of sample. Accordingly, the HD oil was diluted 1:5 in olive oil, while the final 2 µl of EO was mixed with 8 µl of olive oil and applied to the corresponding disc. Notably, no reproducible data were obtained when DMSO was used (data not shown) rather than olive oil as a carrier at the same dilutions (1:5). After maceration with olive oil, the rosemary contents were ready with their carrier, so 10 µl was immediately loaded on the corresponding disc. Finally, the SC-CO_2_ product was dissolved in DMSO, and its volatile oil content was determined.

A variety of essential oils, including rosemary, have been shown to be potent antimicrobial agents. These authors attributed the activity to the contents of major compounds, including 1,8-cineol and camphor^25–27^. Based on our antimicrobial results, both macerated and SC-CO_2_ oils inhibited the growth of the tested bacteria and yeast but not the fungi (Table 5). However, there was differentiation in the recorded inhibition zones. Interestingly, SC-CO_2_ oil showed the best results against the tested panel of bacteria and yeasts. SC-CO_2_ had an approximately 1.5-fold greater inhibitory effect on both the susceptible yeasts *C. albicans* and *C.* tropicalis than did HD oil. Concerning the gram-negative bacteria, SC-CO_2_ oil inhibited the growth of *E. coli* and *S. typhimurium* with inhibition zones ~ 1.5- and 2.0-fold greater than those of standard HD oil, respectively. Moreover, the same oil showed an approximately 2.5-fold increase in the inhibition zone compared to that of HD against the positive bacteria *B. subtilis* and *S. aureus*.

**Table 5:** Antimicrobial properties of rosemary essential oils.

Microorganism	Inhibition zone [Mean ± SEM (mm)]			
	HD/Olive oil (1:5)	OM	SC-CO_2_	Reference
*C. Albicans*	16 ± 0.5	18 ± 1.2	22.5 ± 2.5	22.8 ± 0.7
*C. tropicals*	13 ± 0.0	19 ± 1.2	23.7 ± 2.9	22.2 ± 0.7
*E. Coli*	15 ± 0.0	20 ± 1.5	23.0 ± 1.0	16.8 ± 0.4
*S. typhimurium*	13 ± 0.0	22 ± 1.5	24.0 ± 1.0	16.2 ± 0.4
*B. Subtilis*	10 ± 0.0	15 ± 0.9	27.5 ± 2.5	16.0 ± 0.4
*S. aureus*	12 ± 0.0	16 ± 0.9	29.5 ± 3.5	19.0 ± 0.8
*A. niger*	14 ± 0.0	–	–	21.0 ± 1.1
*M. phaseolina*	15 ± 3.0	–	–	17.4 ± 0.9

SEM, standard error of the mean; mm, millimeter; SC-CO_2_, supercritical carbon dioxide; HD, hydrodistillation; OM, macerated rosemary with olive oil; -, no inhibition zone. All discs were loaded with 10 µl, while 8 µl of olive oil was applied as a carrier control for HD/disc mixed with 2 µl of oil. The stock solution of SC-CO_2_ was 10 mg/ml DMSO, while each disc was loaded with 10 µl of solution equivalent to 100 µg/disc. The displayed inhibition zones were calculated from 4 independent experiments. Reference: For the antibacterial assay, 10 µg/ml amoxicillin trihydrate was used, while 1 mg/ml clotrimazole was applied during the antifungal assays.

The antifungal effect of HD was more pronounced than that of both other oils. This recorded activity could be attributed to the p-cymene^28^, α-terpineol, and 4-terpineol^29^ monoterpenes, as indicated by their antifungal potential. Further synergism could be obtained due to the effects of multiple bioactive agents^30^.

In the present study, maceration was successful because most monoterpenes are lipophilic^31^ and are easily extracted into macerated olive oil. However, three bioactive monoterpenes, p-cymene, 6-terpineol, and 4-terpineol, were not detected under our procedure. The three are already present in small amounts of HD oil (Table 3). Both 6-terpineol and 4-terpineol are isomers of α-terpinol, while the latter is the most abundant isomer^32^. Attention toward oil maceration is important, particularly when the active components are fat soluble. However, it should be well investigated to obtain mostly all of the oil ingredients. Moreover, maceration in olive oil provides an opportunity to fight several disseminated infectious diseases. In addition, it is a household technique that can be applied for several therapeutic purposes, such as cosmetics, skin protection, and massages.

## Conclusion

The chemical profile and bioactivity of rosemary essential oil are affected by the extraction technique. Overall, 70% of the EO oil was extracted by the olive oil in our study. This percentage could be increased by exceeding the shaking intervals and milling the herb to obtain a finer powder. Heating the mixture of rosemary and olive oil at no more than 40 °C could facilitate the diffusion of the volatile ingredients into the olive oil. SC-CO_2_ extraction at 40 °C and 100–300 bar pressure introduced different proportions of volatile components compared with those of HD and OM oils. Despite the obvious antibacterial and antiyeast properties of the SC-CO_2_ and OM oils, neither were able to affect the tested fungi.

## Data Availability

All the data are presented in the manuscript.
